# Ionic Conduction in Polymer‐Based Solid Electrolytes

**DOI:** 10.1002/advs.202201718

**Published:** 2023-01-25

**Authors:** Zhuo Li, Jialong Fu, Xiaoyan Zhou, Siwei Gui, Lu Wei, Hui Yang, Hong Li, Xin Guo

**Affiliations:** ^1^ School of Materials Science and Engineering State Key Laboratory of Material Processing and Die & Mould Technology Huazhong University of Science and Technology Wuhan 430074 P.R. China; ^2^ Department of Mechanics School of Aerospace Engineering Huazhong University of Science and Technology Wuhan 430074 P.R. China; ^3^ Institute of Physics Chinese Academy of Sciences Beijing 100190 P.R. China

**Keywords:** composite polymer electrolyte, interfacial interaction, ionic conduction, polymer electrolyte, solid‐state batteries

## Abstract

Good safety, high interfacial compatibility, low cost, and facile processability make polymer‐based solid electrolytes promising materials for next‐generation batteries. Key issues related to polymer‐based solid electrolytes, such as synthesis methods, ionic conductivity, and battery architecture, are investigated in past decades. However, mechanistic understanding of the ionic conduction is still lacking, which impedes the design and optimization of polymer‐based solid electrolytes. In this review, the ionic conduction mechanisms and optimization strategies of polymer‐based solid electrolytes, including solvent‐free polymer electrolytes, composite polymer electrolytes, and quasi‐solid/gel polymer electrolytes, are summarized and evaluated. Challenges and strategies for enhancing the ionic conductivity are elaborated, while the ion‐pair dissociation, ion mobility, polymer relaxation, and interactions at polymer/filler interfaces are highlighted. This comprehensive review is especially pertinent for the targeted enhancement of the Li‐ion conductivity of polymer‐based solid electrolytes.

## Graphical abstract

This article reviews the ionic conduction mechanisms and optimization strategies of polymer‐based solid electrolytes, including solvent‐free polymer electrolytes, composite polymer electrolytes, and quasi‐solid/gel polymer electrolytes, while the ion‐pair dissociation, ion mobility, polymer relaxation, and interactions at polymer/filler interfaces are highlighted.

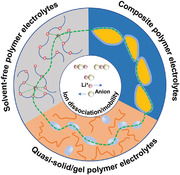

## 1 Introduction

Commercial liquid electrolytes are associated with safety concerns because of their flammability and leakage.^[^ ^1^ ^]^ Solid electrolytes are inherently safe, promising to overcome safety issues of Li‐ion batteries.^[^ ^2^ ^]^ Moreover, solid electrolytes are stable with metallic Li anodes and exhibit potential to inhibit the Li dendrite growth. Therefore, solid electrolytes enable high energy density in solid‐state batteries when using Li anodes.^[^ ^3^ ^]^

Solid electrolytes can be roughly classified into two classes: ceramics and polymers.^[^ ^4^ ^]^ Compared with ceramic electrolytes, polymers have several advantages, including easy synthesis, low mass density, low cost, large‐scale manufacturing process compatibility, and high mechanical toughness.^[^ ^5^ ^]^ Thus, polymer‐based electrolytes are very promising for solid‐state batteries.^[^ ^6^ ^]^ However, despite big advances achieved, polymers such as polyethylene oxide (PEO) still exhibit an ionic conductivity significantly lower than their liquid or ceramic counterparts.^[^ ^7^ ^]^ For a good solid electrolyte, high ionic conductivity is of paramount importance;^[^ ^8^ ^]^ there is always immense interest in improving the ionic conductivity of electrolytes without negatively affecting other properties, such as mechanical strength.

Understanding the mechanisms for the fast ionic conduction and the quantitative prediction of the ionic conductivity is extremely important for the design and development of polymer‐based solid electrolytes. Numerous studies have attempted to elucidate mechanisms of the ionic conduction in polymer electrolytes.^[^ ^9^ ^]^ Guo et al.^[^ ^10^ ^]^ studied the ionic conduction mechanism in composite polymer electrolytes by electrochemical methods and Monte–Carlo simulations, reporting that the space‐charge regions at the two‐phase interfaces dominate the ionic conduction. Canesa et al.^[^ ^11^ ^]^ investigated the effect of SiO_2_ nanoparticles on the ion‐transport in PEO/LiBF_4_/SiO_2_ composite polymer electrolytes by the molecular dynamics simulation, revealing that the nanoparticle addition slowed down the dynamics of polymer segmental motion; thus, decreasing the overall ionic conductivity of the electrolyte. Borodin et al.^[^ ^12^ ^]^ carried out molecular‐dynamic and Monte–Carlo simulations for PEO‐based electrolytes, and explained the charge migration in the system in terms of the renewal of hopping probabilities. Hu et al.^[^ ^13^ ^]^ studied the ionic conduction pathways in PEO/Li_7_La_3_Zr_2_O_12_ (LLZO) systems by combining selective isotope labeling and solid‐state nuclear magnetic resonance (ssNMR), indicating that Li ions favor pathways through the ceramic phase.

Previous works report the enhancement of the ionic conductivity of polymer‐based solid electrolytes by designing polymer hosts,^[^ ^14^ ^]^ modifying ceramic fillers,^[^ ^15^ ^]^ and introducing additives.^[^ ^16^ ^]^ Based on intermolecular interactions, Cui's group^[^ ^17^ ^]^ proposed intermolecular chemistry for developing polymer electrolytes with acceptable ionic conductivity and high mechanical strength. Lin et al.^[^ ^18^ ^]^ developed a polycarbonate‐based electrolyte with increased polar groups, the ionic conductivity of which was significantly increased. Bae et al.^[^ ^19^ ^]^ designed nanostructured Li_3_ *_x_* La_2/3−_ *_x_* TiO_3_ (LLTO) fillers to construct three‐dimensional (3D) continuous conducting networks in composite polymer electrolytes. Lin et al.^[^ ^20^ ^]^ developed a modified silyl‐terminated polyether‐based polymer electrolyte with a 3D network structure, and achieved an improved ionic conductivity of 3.6 × 10^−4^ S cm^−1^. However, polymer‐based electrolytes with high ionic conductivities often have compromised mechanical strength.^[^ ^5^ ^]^ To develop polymer‐based electrolytes with high ionic conductivity and mechanical strength is very challenging; most studies on optimizing the properties of polymer‐based electrolytes attempted to balance the mechanical strength and the ionic conductivity.^[^ ^21^ ^]^

In this review, recent progresses in the research of polymer‐based solid electrolytes, including solvent‐free solid polymers, composite polymer electrolytes, and quasi‐solid/gel polymers are assessed; current understanding of the ionic conduction, and challenges and strategies for enhancing the ionic conduction are discussed. This comprehensive review will be helpful for the targeted improvement of the ionic conductivity of polymer‐based solid electrolytes. Although the overall ionic conduction in polymers, including conductions of cations and anions, is discussed, the focus of this article is on the Li‐ion conduction.

## 2 Solvent‐Free Polymer Electrolytes

In polymer electrolytes, lithium salts are dissolved in polymers to create cations and anions. Lithium salts, for example, LiN(CF_3_SO_2_)_2_ (LiTFSI),^[^ ^22^ ^]^ LiN(FSO_2_)_2_ (LiFSI),^[^ ^23^ ^]^ LiCF_3_SO_3_,^[^ ^24^ ^]^ and LiClO_4_,^[^ ^25^ ^]^ are dissolved in a polymer matrix to provide the ionic conduction. Polymers should fulfill some essential criteria: the ability to dissolve lithium salts and form polymer‐salt complexes, chemical/electrochemical stability, and physical supportability.^[^ ^26^ ^]^ Numerous polymers, for example, polyacrylonitrile (PAN),^[^ ^27^ ^]^ poly(formaldehyde) (POM),^[^ ^28^ ^]^ Poly(Vinylene Carbonate) (PVC),^[^ ^29^ ^]^ polymethyl methacrylate (PMMA),^[^ ^30^ ^]^ polyvinylidene fluoride (PVDF),^[^ ^31^ ^]^ polypropyl carbonate (PPC),^[^ ^32^ ^]^ and PEO,^[^ ^33^ ^]^ have been used in polymer electrolytes. Among them, PEO and its derivatives are most commonly used, because of their low cost, acceptable mechanical stability, good electrode compatibility, high film‐forming capability, and ion‐transport facilitation (high donor number for Li^+^).^[^ ^34^ ^]^ However, polymers generally present ionic conductivities lower than 10^−6^ S cm^−1^ at room temperature.^[^ ^7^ ^]^

Polymer electrolytes are complexes formed by reactions of alkali metal salts with polar or Lewis‐acid–base active groups (e.g., —C=O, —C—O—, —P—, —N—, —S—, and —C≡N) of polymer hosts. One then expects a close relationship between the ability to form homogeneous complexes and the ability of monomers to dissolve salts as well as dissociate with Li ions. Such complex reactions are thermodynamically favorable only if the Gibbs energy of the salt solvation in polymers is large enough to overcome the lattice energy of the corresponding salt. In addition to the very important lattice energy consideration, a number of other criteria that determine the possibility of forming complexes are: i) a high concentration of polar (basic) groups on the polymer chain is needed to solvate salts effectively, ii) the cohesive energy of the polymer cannot be too high, and its flexibility, as indicated by a low glass‐transition temperature, should be quite high, so that reorientation of the local coordination geometry to achieve effective solvation, may be achieved.^[^ ^35^ ^]^ Generally, the higher the concentration of polar groups of polymers and/or the lower the lattice energy of added salts, the higher the charge carrier concentration.^[^ ^36^ ^]^

The dissociation energy of Li‐ions with active groups is the key factor for the Li^+^ transport in polymer chains, and may be related to the Li^+^ coordination number as well as interactions between Li^+^ and active groups.^[^ ^37^ ^]^ Most polymer electrolytes are based on oxygen‐containing monomers, including ethers in poly(ethylene oxide) and poly(propylene oxide), while other Lewis‐base groups have also been employed, including nitrogen in poly(ethylenimine). In general, Lewis‐base groups on the complexing host species are required to coordinate cations of salts; and thus, produce a favorable Gibbs energy for the polymer‐salt interactions. However, in PEO or Lewis‐base polymers, Li^+^ ions strongly interact with active groups to cause cation–polymer interactions, which leads to relatively sluggish Li‐ion diffusion and rapid anion diffusion. Replacing these polymers with a Lewis‐acidic polymer (e.g., —C—S— group based polymer, poly(thioethers)) can reverse this relationship, eventually leading to an increase in the Li^+^ diffusion while preserving the same salt solubility.

### 2.1 Ionic Conduction

The ionic conduction in polymer electrolytes, which usually features multiphase structures at microscopic and/or macroscopic levels, is very complex.^[^ ^38^ ^]^ First, the coexistence of different phases, such as amorphous phases and various crystalline complexes of PEO and Li^+^, provides different pathways for the ionic conduction; second, the distribution and structure of phases are intricate. A number of experimental and theoretical studies have identified a variety of relevant transport mechanisms, such as cation hopping through the formation of a weak coordination shell between Li^+^ ions and ether oxygens (EO), and free ion transport along percolating channels in the PEO melt.^[^ ^11^, ^39^ ^]^ However, among different controlling factors, the segmental motion of the polymer backbone has been identified as a key factor for the cation and anion mobilities.

**Figure 1:**
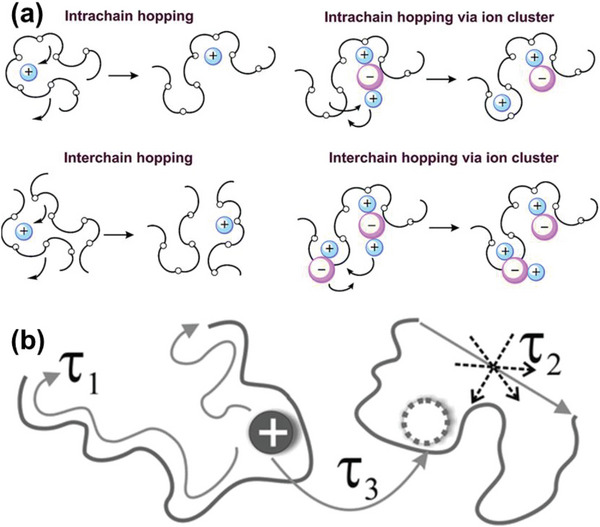
a) Li‐ion conduction occurring in the amorphous region of polymer. Reproduced with permission.^[^ ^6b^ ^]^ Copyright 2015, Royal Society of Chemistry. b) Li‐ion transport arising from intra‐chain motion, polymer‐segment relaxation, and inter‐chain hopping. Reproduced with permission.^[^ ^41^ ^]^ Copyright 2007, American Physical Society.

It is rather well established that the ionic conduction in polymer electrolytes includes local motion of polymer segments, inter‐ and intra‐chain ion‐hopping between coordinating sites (**Figure** **1**a),^[^ ^6^, ^40^ ^]^ which, as mentioned above, are not fixed and vary with time and temperature. Thus, the ionic conduction has three primary contributors: inter‐chain motion of ions along a chain (*τ*_1_), polymer‐segment relaxation (*τ*_2_), and intra‐chain hopping from one chain to another (*τ*_3_), as illustrated in Figure 1b.^[^ ^41^ ^]^ In the case of PEO, Li^+^ ions are coordinated by ether oxygen atoms of the PEO chains and move via the coupling/decoupling of Li—oxygen bonds. Similar ionic conduction mechanism is also exhibited in other polymers with polar groups, such as PAN (containing C≡N), with which it is easy to form a polymer/salt complex.^[^ ^4^, ^29^, ^39^, ^42^ ^]^

**Figure 2:**
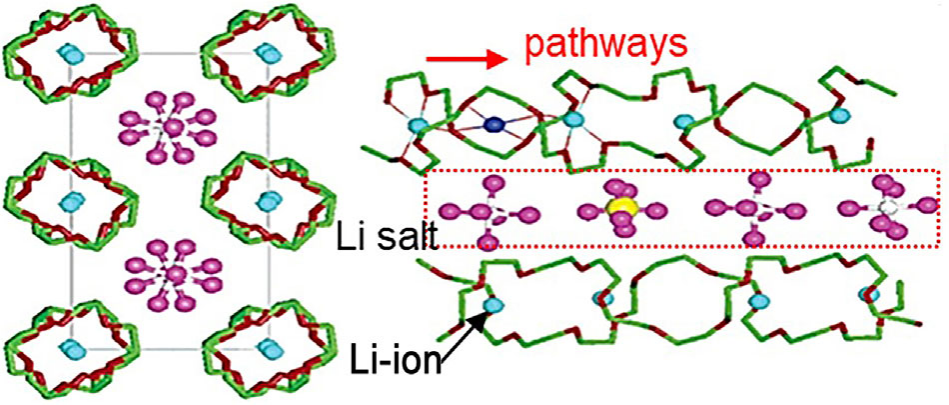
Li‐ion conduction occurring through the crystalline region in polymer. Reproduced with permission.^[^ ^45^ ^]^ Copyright 1999, Springer Nature.

Segmental motion cannot, by definition, occur in crystalline complexes and, as a result, such crystalline ion–polymer complexes were believed to be insulators.^[^ ^43^ ^]^ Recently; however, this statement has been overturned; ionic conductivity has also been reported in the crystalline domains of a polymer electrolyte and has been argued to be higher than that in the amorphous phase. Bruce et al.^[^ ^44^ ^]^ discovered ion‐conducting crystalline ion–polyether complexes and proposed a mechanism for the ion transport. Through comprehensive research of crystalline PEO_6_/Li*X*F_6_ (*X* = P, As, Sb) polymer electrolytes, Bruce et al.^[^ ^45^ ^]^ reported a novel Li‐ion migration mechanism in the crystalline region of PEO‐based electrolytes: Two crystalline PEO chains folded to form a cylindrical tunnel, within which Li ions were coordinated by ether oxygen, while anions were located outside the tunnel in the inter‐chain space, as shown in **Figure** **2**; Li ions migrated from one site to another along the cylindrical tunnel without the aid of the segmental motion. Nevertheless, following debate, experimental and theoretical studies contradict that the conductivity is higher in the crystalline domains, and there is now a consensus that the ionic transport occurs predominantly in amorphous polymers rather than the crystalline phase.^[^ ^43^, ^46^ ^]^

**Figure 3:**
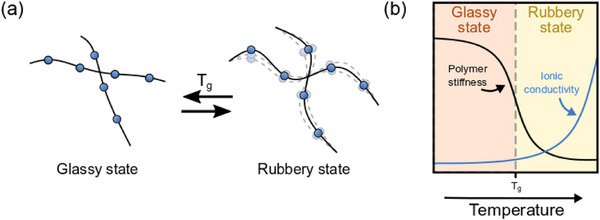
a) Schematic representation of the behavior of polymer segments above and below the glass transition temperature and b) relationship of polymer stiffness and ionic conductivity as a function of temperature. Above *T*_g_, polymer segments become more flexible, which aids the ionic conduction. Reproduced with permission.^[^ ^47^ ^]^ Copyright 2021, Elsevier.

Amorphous polymers exhibit transitions from “glassy” to “rubbery” states as they are heated, and vice versa when cooled back down. In the glassy state, they are brittle with rigid polymer strands; in the rubbery state, polymer chains become more flexible (**Figure** **3**a).^[^ ^47^ ^]^ This transition takes place at the glass transition temperature *T*_g_, which is one of the central material properties of solid polymer electrolytes. Polymer chain mobility is critical for the ionic conduction. Accordingly, below the glass transition temperature, where polymer chains are largely rigid and immobile, amorphous polymers have a near‐zero ionic conductivity. Therefore, solid polymer electrolytes conduct very poorly near or below their glass transition temperatures; above *T*_g_, the local polymer chain motion is in fact liquid‐like and rapid (Figure 3b). It is important to note that only the amorphous phases of polymers experience glass transitions; the crystalline phases melt instead.

One very important concept in the mechanistic understanding of polymer ionics is the issue of coupling between transport and relaxation.^[^ ^48^ ^]^ When the host polymer relaxes more rapidly, the ionic conductivity increases. This was formalized by Angell,^[^ ^49^ ^]^ with the use of a decoupling ratio (*R*_τ_), defined by

(1) Rτ=τsτsτστσ

where *τ*_s_ is the structural relaxation time that refers to viscosity or segmental relaxation, while *τ*_*σ*_ is the conductivity relaxation time (inversely proportional to the conductivity), which can be determined from the DC conductivity *σ*_dc_,

(2) τσ=εαe0εαe0σdc≈10−1210−12σdcσdcσdc≈10−1210−12σdcσdc

where the permittivity of vacuum *e*_0_= 8.85 × 10^−14^ F cm^−1^, and optical dielectric constant *ε*_*α*_ ≈ 12.^[^ ^43^ ^]^ Equation (2) generally provides an approximation of the conductivity relaxation time. One of the ways to experimentally determine the conductivity relaxation time is to use the maximum of the dielectric modulus or the dielectric relaxation frequency.^[^ ^48^ ^]^

The decoupling index is indicative of a very close relationship between the structural relaxation process (due to chain motion and reflected in *τ*_s_) and the conductivity (inversely proportional to *τ*_*σ*_). For glassy electrolytes below the glass‐transition temperature, the structural relaxation time becomes very long, and the decoupling ratio approaches an order of 10^13^ (because *τ*_s_ can be of the order of 200 s at *T*_g_).^[^ ^43^, ^50^ ^]^ When polymer/salt complexes are studied above the glass‐transition temperature, the decoupling ratio generally approaches or is slightly smaller than unity. If *R*_*τ*_ is close to unity, the ionic motion and the structural relaxation occur on the same time scale, suggesting that their rate‐determining steps are the same. If *R*_*τ*_ is substantially less than unity (e.g., *R*_*τ*_ ≅ 10^−3^), the ionic polymer/salt complexes are referred to a strong residual ion–ion coupling, resulting in reduced conduction. As the polymer/salt complex cools toward the glass‐transition temperature, the structural relaxation is slowed and then arrested (the decoupling slowly increases with decrease of *T*); and therefore, the ionic conductivity decreases rapidly.^[^ ^51^ ^]^

The substantial change in the value of *R*_*τ*_, from 10^13^ in glasses to near unity in soft polymer electrolytes must in part be due to the low frequencies and large amplitudes associated with the polymer segment motion in the elastomeric phase above *T*_g_. Generally, in an ionic conducting glass, only small changes in the local geometry are associated with the ionic motion into a vacancy as in a covalent crystal. In polymer materials, in contrast, very large changes in the local geometry are brought about upon complexation of a cation. Subsequently, for the cation to move, the segment complexing must first exchange the primary coordinating atoms, and such motions require segmental mobility.

**Figure 4:**
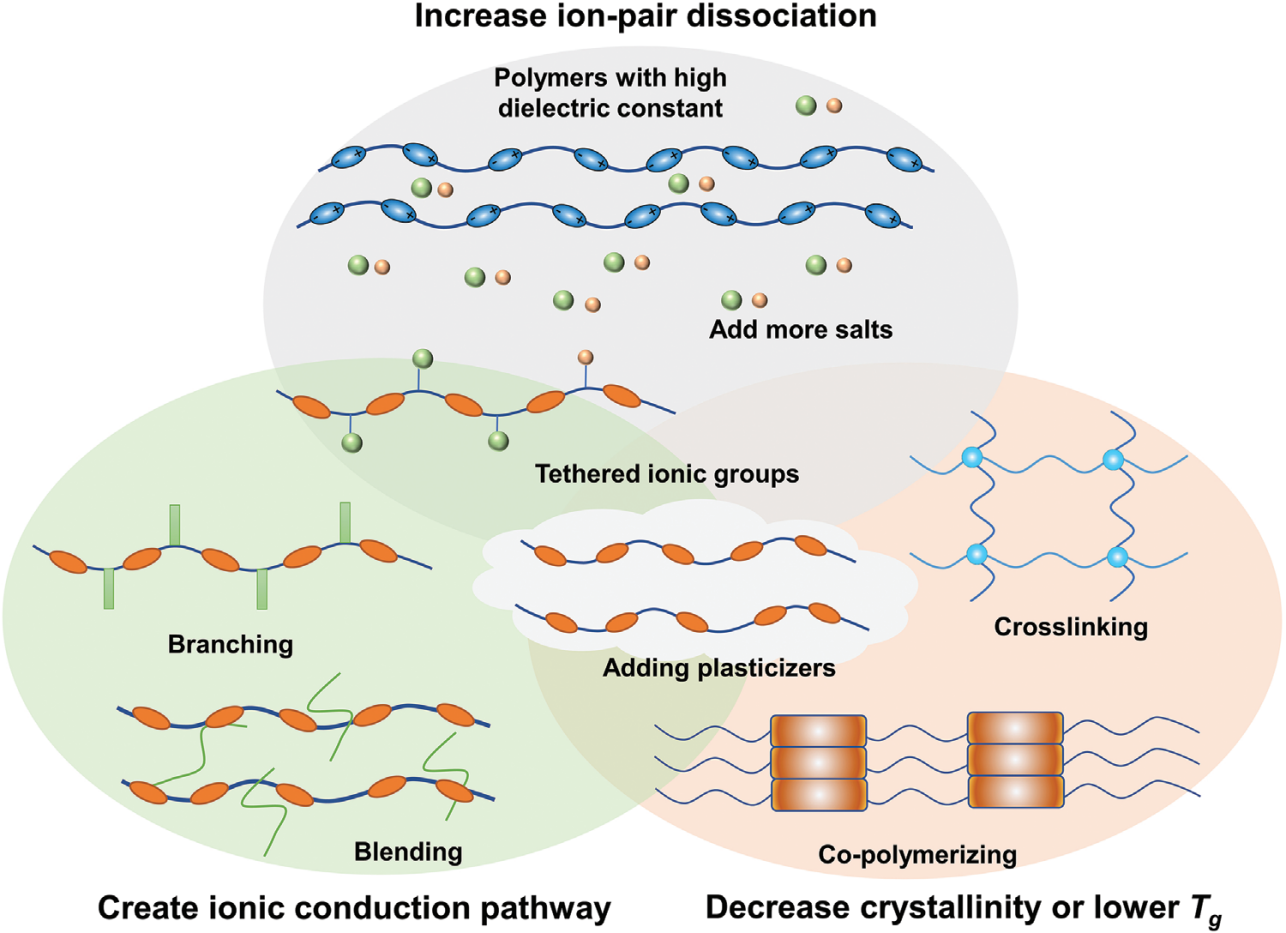
Approaches toward highly ionic‐conducting solid polymer electrolytes.

Efficient Li‐ion transport is related to the local relaxation and segmental motion of polymer hosts, and it is only the thermal energy in excess of the glass‐transition temperature that provides the actual mobility of the local polymer chain segments. The above concepts suggest three ways to enhance the ionic conduction. The first is simply the addition of more salts; thus, increasing the number of mobile ions. The second way is to lower *T*_g_, thus facilitating relaxation and, therefore, the ionic conduction. The third way to increase the conductivity would be to decouple diffusion from relaxation, that is, to create structures in which ions can move without the aid of the host–polymer relaxation; in other words, to create static pathways for the ionic conduction. Main approaches toward highly ionic‐conducting solid polymer electrolytes are presented in **Figure** **4**.

### 2.2 Interpretation of the Ionic Conductivity

Ionic conduction in electrolytes is driven by chemical and electrochemical potential gradients. In a dilute solution, it is fair to assume that all charge carriers are in fact available;^[^ ^48^ ^]^ so that, the conductivity can be described by the Kohlrausch summation:

(3) $$\sigma =\sum_{i}\mu_{i}q_{i}n_{i}$$

and the Nernst–Einstein relationship

(4) σ=niq2niq2kBTkBTD

where the variables *σ*, *µ*_i_,*q*_i_, *n*_i_, *D*, *k*_B_, and *T* are the conductivity, mobility, charge, concentration of the the ion, diffusion coefficient, Boltzmann's constant, and Kelvin temperature, respectively. Although Equations (3) and (4) fail to hold quantitatively in concentrated electrolytes, they still give important indications of how to understand, and thereby optimize the ionic conduction. One would like to increase both the concentration (ion‐pair dissociation) and the diffusion coefficient (mobility) of the mobile ions.

A good ionic conductor must; therefore, be able to simultaneously facilitate the ion‐pair dissociation and exhibit minimal resistance to the ion motion. Although the migration of both cations and anions contribute to the total current, the useful fraction of the current that drives redox reactions at electrodes in most electrochemical cells is carried by cations. The cation transference number characterizes this fraction, and maximizing this number is key to increasing the efficiency of battery operation. A high cation transference number indicates that the conductivity primarily depends on the Li‐ion transport rather than on the anionic mobility. Anion mobility does not contribute to energy storage and represents “wasted” energy.^[^ ^52^ ^]^ At a low cation transference number, local polarization is severe and makes cation deposition uneven.^[^ ^5^, ^53^ ^]^ As a result, the cycle life and power density of the battery are degraded. In addition, anions are not consumed at electrodes; they can build up over time, which may eventually result in their decomposition with detrimental effects for the cell.^[^ ^54^ ^]^ Therefore, an electrolyte with a high cation transference number can exhibit fast charge–discharge capability even with a relatively low ionic conductivity, which suppresses Li dendrites and results in long cycling lifetime with the Li metal anode. The higher the cation transference number of an electrolyte, the better the battery performance is. Overall, the ideal cation transference number is equal to 1.

The ionic mobility and dissociation can be optimized by selecting the used Li‐salt. In polymer electrolytes, the most widely used Li‐salts are LiClO_4_, LiBF_4_, LiPF_6_, LiAsF_6_, LiCF_3_SO_3_, LiTFSI, and so on. The mobility of ions, their dissociation constants and solubility are in the following orders:^[^ ^4^, ^55^ ^]^

Mobility of ion: LiBF_4_ > LiClO_4_ > LiPF_6_ > LiAsF_6_ > LiCF_3_SO_3_ > LiTFSI

Dissociation constant: LiTFSI > LiAsF_6_ > LiPF_6_ >LiClO_4_ > LiBF_4_ > LiCF_3_SO_3_

Solubility: LiTFSI > LiPF_6_ > LiAsF_6_ > LiBF_4_

Generally, salts with larger anions can easily dissolve and dissociate in the PEO matrix and set off free Li cations, resulting in increased ionic conductivity; in this sense, LiTFSI is very promising.

Dissociated ions that overcome the energy barrier hop from one site to another in a solid polymer electrolyte.^[^ ^56^ ^]^ In most polymer electrolytes, the ion‐pair dissociation and the polymer relaxation are usually thermally activated. Therefore, the relationship between the electrical conductivity, *σ*, and temperature is explained in accordance with the well‐known Arrhenius model, expressed by Equation (5):^[^ ^57^ ^]^

(5) $$\sigma =\sigma_{0}\exp\left(\frac{-E_{a}}{k_{B}T}\right)$$

where *σ*_0_ is the pre‐exponential factor and *E*_a_ is the activation energy for conductivity. According to the Arrhenius equation, the plot of log*σ* versus 1/1TT features a typical slope; materials that exhibit the linear Arrhenius variation indicate that the ionic conduction occurs via a simple hopping mechanism decoupled from polymer chain breathing (e.g., the cases of crystalline polymers below *T*_g_, ceramic ion conductors, etc.).^[^ ^9^ ^]^

Above the glass‐transition temperature (*T*_g_), individual units can move around their bonding points and assist the ion migration. Thus, at temperatures above *T*_g_, the ionic motion is coupled to structural relaxations. These relaxations are dependent on the viscosity of the system, which decreases as the temperature increases above *T*_g_. The Vogel–Tammann–Fulcher (VTF) equation captures this effect by coupling the ionic conductivity to the difference in temperature from the ideal glass transition. The VTF behavior is described by Equation (6):^[^ ^57^ ^]^

(6) $$\sigma =\sigma_{0}T^{-1/2}\exp\left(-\frac{B}{T-T_{0}}\right)$$

where *B* is the pseudo‐activation energy for conductivity (expressed in unit of $E_{a}/k$), and *T*_0_ is the reference temperature, which normally falls 10 to 50 K below the experimental (kinetic) glass‐transition temperature. The VTF equation was devised for describing the diffusion process in glassy and disordered materials, mainly used for describing the ionic conduction in gel polymers and solid polymer electrolytes above *T*_g_ of polymer hosts.

However, for some low‐melting polymers such as PEO, plots of *σ* versus 1/T are typically nonlinear, indicative of a conduction mechanism that involves the ionic hopping coupled with the relaxation/breathing and/or segmental motion of polymeric chains, which can be modeled in terms of a combination of Arrhenius and/or VTF behaviors. The Arrhenius and VTF equations are commonly used to analyze the ionic conduction in polymer electrolytes.^[^ ^58^ ^]^

The conductivity of composite materials consisting of a conducting and an insulating phase may be described, in principle, by effective medium theories.^[^ ^57^ ^]^ Most aspects of general percolation and effective medium theories can be combined to give the general effective medium (GEM) equation:

(7) $$\frac{f\left(\sigma_{1}^{1/t}-\sigma_{m}^{1/t}\right)}{\sigma_{1}^{1/t}+A\sigma_{m}^{-1/t}}+\frac{\left(1-f\right)\left(\sigma_{2}^{1/t}+\sigma_{m}^{1/t}\right)}{\sigma_{2}^{1/t}+A\sigma_{m}^{1/t}}=0$$

where *σ*_1_, *σ*_2_, and *σ*_m_ are the conductivities of the two individual phases and the composite material, respectively, the constant *A* depends on the particular composite medium and the approach to the problem, and the exponent *t* is related to the filler volume fraction *f* and to the grain shape. The effective medium theory nicely describes the ionic conduction in composite polymer electrolytes consisting of a polymer matrix and fillers, indicating that the enhanced ionic conductivity is caused by interfacial interactions of the polymer and the fillers (space‐charge effect),^[^ ^59^ ^]^ which is discussed in the later part of this article.

In fact, it is the diffusivity or mobility rather than the conductivity of ions that might be related to the polymer chain motions. According to Equations (3) and (4), the conductivity depends on not only mobility but also on the concentration of charge carriers. In dilute solutions, it is assumed that all charge carriers are available, so that the number *n* in Equations (3) and (4) is simply the stoichiometric number of ions. However, most polymer electrolytes are not dilute solutions; then, Coulombic interactions among charge carriers are crucial for determining the ionic conductivity.^[^ ^35^ ^]^ Under these conditions, ions do not move freely; and therefore, the concentration of charge carriers, *n*, is dependent on the temperature, the stoichiometric concentration, and the physical properties of the polymer host. Watanabe and co‐workers^[^ ^60^ ^]^ suggested that, as the salt concentration increases starting from a dilute complex, the conductivity first increases and then, after attaining a maximum for a particular concentration, falls off quickly for more concentrated materials. This phenomenon was confirmed by Paillard,^[^ ^61^ ^]^ whose results are listed in **Table** **1**. At low concentrations, the charge carrier number increases as the salt is added, so that mobile‐ion number increases as does the ionic conductivity. At higher concentrations, the salt acts as a weak sort of cross‐linker, and vibrational spectroscopic investigation clearly demonstrates the formation of ionic pairs or/and ionic clusters.^[^ ^62^ ^]^ Ionic pairs cannot contribute to the ionic conductivity due to electrical neutrality, while ionic clusters are too big to move.^[^ ^63^ ^]^ Moreover, the formation of ionic pairs or ionic clusters impedes the mobility of polymer chains, reducing the ionic conductivity and the cation transference number.

**Table 1:** Cation transference numbers and ionic conductivities of PEO‐based polymer electrolytes with different Li salt contents.^[^ ^61^ ^]^

Concentration (O/Li)	Transference number	Ionic conductivity [×10^−4^ S cm^−1^, @70 °C]
34.8	0.25	2.1
23.2	0.20	2.9
13.4	0.19	2.2

### 2.3 Ionic Conductivity Versus Mechanical Strength

As discussed in Section 2.2, the ionic conduction occurs primarily in the amorphous phase; so, the partial crystallinity of most PEO complexes is an unwelcome complication when studying the conductivity response of materials. As discussed in the Figure 4, the *T*_g_, crystallinity, and ion‐pair dissociation remain the most crucial parameters to consider when designing polymer electrolytes with high conductivity. Plasticizers are materials composed of weakly interacting molecules that provide a high‐entropy medium for the ion migration to decrease the crystallinity and increase the free volume.^[^ ^64^ ^]^ Plasticizers also promote the dissociation of ion pairs and, as a result, increase the number of free Li^+^ ions available for the charge transport. However, introducing plasticizers often results in a liquid‐like mechanical behavior of polymers; this tradeoff coupling of the conductivity and the mechanical strength leads to a serious safety concern for the applications of polymer electrolytes.

To develop a polymer electrolyte simultaneously featuring high ionic conductivity and mechanical strength is very challenging.^[^ ^17^ ^]^ Molecular‐architecturally engineered polymers can combine merits of two or more polymers, while eliminating weaknesses of individuals.^[^ ^65^ ^]^ For example, co‐polymerized polymers, formed by co‐polymerization of two or more different monomers into ordered supramolecular structures, effectively incorporate the merits of polymers with different characteristics, of which the flexible one acts as the ionic conductor, while the co‐polymerized parts impart the electrolytes with other desired properties, such as good mechanical strength and electrochemical stability; the structure of co‐polymerized polymers is shown in Figure 4.^[^ ^6^, ^66^ ^]^ Therefore, molecular architectural engineering can achieve a remarkable ionic conductivity without sacrificing other properties (mechanical strength and electrochemical stability) of polymer electrolytes. Specifically, polymers with new molecular architectures can interrupt repeating units; thus, preventing crystallization.^[^ ^67^ ^]^ A general concept for the molecular architectural engineering is to utilize a flexible backbone to which short‐chain polar oligomers capable of complexing alkali metal salts are attached, to accelerate the local thermal motion of polymer segments and relaxation of polymer segments.^[^ ^68^ ^]^

Typical polymer molecular structures are: cross‐linked polymers, co‐polymerized polymers, block polymers, comb‐like polymers, branched polymers, and blended polymers.^[^ ^54^ ^]^ For example, Cui et al.^[^ ^69^ ^]^ introduced nanoporous polyimide (PI) into the PEO/LiTFSI system to build a blending polymer electrolyte. The robust and nonflammable PI can provide vertically aligned nanochannels for the ion transport; thus, the ionic conductivity of the polymer electrolyte can be up to 2.3 × 10^−4^ S cm^−1^ at 30 °C. Bouchet et al.^[^ ^70^ ^]^ reported a triblock copolymer electrolyte based on modified linear PEO central block with two polystyrene (PS) lateral blocks by the controlled radical polymerization from PS functional units. Chemical defects that are homogeneously distributed along the PEO chains can break the stereo‐regularity and decrease the melting temperature and crystallinity of polymer; and thus, the ionic conductivity is up to 1.3 × 10^−5^ S cm^−1^ at 60 °C, and the cation transport number reaches values greater than 0.85. Besides, the introduction of lateral PS functional units has ensured the mechanical property of the PEO electrolyte.

## 3 Composite Polymer Electrolytes

Dispersing inorganic fillers in polymers to prepare composite polymer electrolytes is one of the most effective ways to increase the ionic conductivity as well as the mechanical strength and/or electrochemical stability. Composite polymer electrolytes integrate advantages of organic and inorganic materials, while mitigating their disadvantages. Ionic conductivity of composites can be increased by up to two orders of magnitude (increase from 10^−6^ to 10^−4^ S cm^−1^), as compared with pure polymers.^[^ ^71^ ^]^

### 3.1 Roles of Fillers

Commonly used fillers in composite polymer electrolytes are broadly classified into two categories: inert fillers, such as TiO_2_,^[^ ^71a^ ^]^ SiO_2_,^[^ ^72^ ^]^ Al_2_O_3_,^[^ ^73^ ^]^ ZnO_2_,^[^ ^74^ ^]^ palygorskite,^[^ ^75^ ^]^ nanoclay;^[^ ^76^ ^]^ and active fillers (Li‐ion conductive), including Li_10_GeP_2_S_12_ (LGPS),^[^ ^77^ ^]^ LLZO,^[^ ^78^ ^]^ LLTO,^[^ ^79^ ^]^ Li_1.4_Al_0.4_Ti_1.6_(PO_4_)_3_ (LATP),^[^ ^80^ ^]^ and Li‐ion conducting MOFs.^[^ ^81^ ^]^ The original idea about filler's roles is to decrease the crystallinity and increase the amorphous phase content of polymers. Subsequent reports indicate that the addition of fillers not only prevents the polymer crystallization but also promotes specific interactions among surface groups and interfacial effects.^[^ ^82^ **^]^**

#### 3.1.1 Inert Fillers

**Figure 5:**
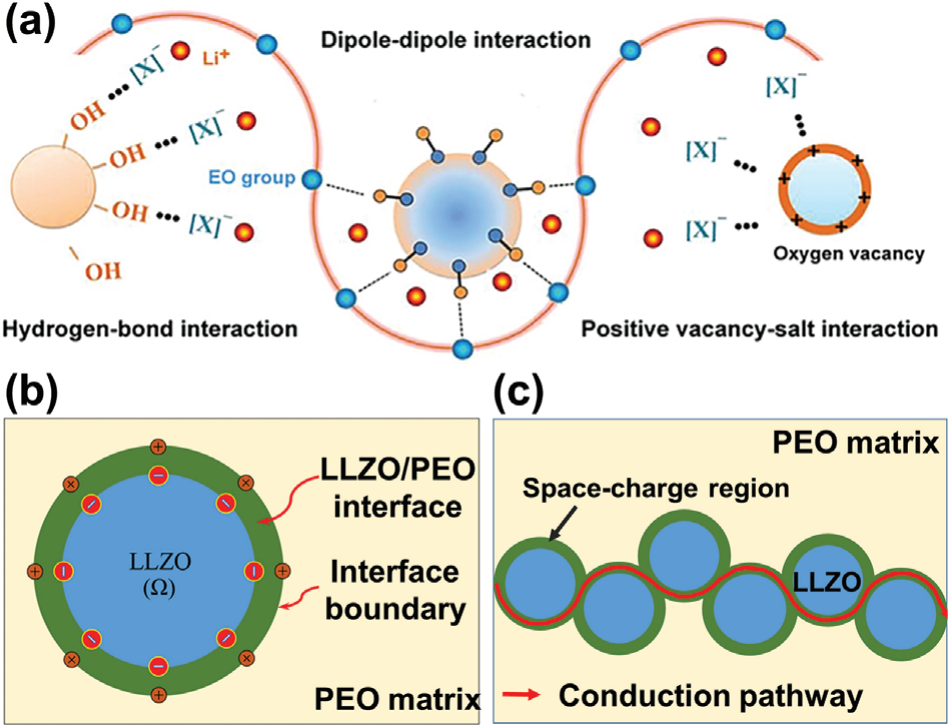
a) Illustration of Lewis acid–base interactions between fillers and polymer hosts. Reproduced with permission.^[^ ^17^ ^]^ Copyright 2019, John Wiley and Sons. b) Space‐charge region between polymer host and filler and c) space‐charge regions providing pathway for fast ionic conduction. Reproduced with permission.^[^ ^10^ ^]^ Copyright 2019, American Chemical Society.

Generally, inert inorganic nanofillers are analogous to molecular plasticizers, which may increase the free volume in the polymer matrix and speed up the segmental dynamics; thus, inhibiting the polymer crystallization and decreasing *T*_g_. Inert fillers are usually Lewis acid or base centers, and it is easy to induce the Lewis acid–base interactions between fillers and polymers. Croce et al.^[^ ^83^ ^]^ suggested that inert fillers played two important roles in the polymer matrix (**Figure** **5**a). First, they were cross‐linking centers for PEO segments and Li‐salt anions, modifying the interfacial structure and creating pathways for transporting Li^+^ independent of the segmental motion. Second, they were centers of Lewis acid–base interactions for ionic species; thus, reducing the ionic coupling and promoting the salt dissociations by forming “ion‐ceramic complexes.” However, poorly dispersed inert inorganic fillers can oppositely affect the ionic conduction by serving as excess cross‐linking sites for polymer chains with polar groups and anions, which reduce the segmental dynamics, ultimately reducing the ionic mobility and conductivity.

#### 3.1.2 Active Fillers

Compared with inert fillers, active fillers play some additional roles, thereby contributing a higher ionic conductivity.^[^ ^84^ ^]^ Active fillers are more likely to reconstruct the interfaces between the filler particles and the polymer matrix. It is believed that the conductivity enhancement is mainly attributed to the percolation across the interfaces, and the interfacial regions can easily expand to twice the particle radius.^[^ ^85^ ^]^ Guo et al.^[^ ^10^ ^]^ suggested that adding active fillers could induce the formation of space‐charge regions at polymer/filler interfaces, resulting in the accumulation of Li‐ions on one side of the interface (Figure 5b). When the generated space‐charge regions in individual nanoparticles were connected to each other, Li‐ion transport expressways were formed (Figure 5c); then, the ionic conduction was significantly improved. In addition, appropriate amount of active fillers can form connected conductive networks to support the Li^+^ diffusion through the fillers. However, a further increase in the amount of active inorganic fillers also leads to a lower ionic conductivity, which can be attributed to their irregular agglomeration effect.

### 3.2 Interactions at Polymer/Filler Interfaces

Many researchers suggested that the ionic conductivity enhancement in composite polymer electrolytes originates from the interfacial interactions between ceramic fillers and polymer matrix.^[^ ^86^ ^]^ As discussed in Section 3.1, introducing a secondary phase into the matrix creates interfacial interactions between the polymer and the fillers, which offer a distinctive venue for the ionic conduction. The thermodynamics at the interfaces can be described by the Lewis acid–base effect and the space‐charge effect.

#### 3.2.1 Lewis Acid–Base Effect

Lewis acid–base effects are important factors affecting the ionic conductivity. A relevant model was proposed by Wieczorek in 1996 to explain the ionic conduction in the PAN‐LiClO_4_/Al_2_O_3_‐system.^[^ ^91^ ^]^ Fillers with the Lewis acid–base character compete with Li‐ions to interact with polymer chains, helping to separate ion pairs and increase the concentration of free Li‐ions. Lewis acid–base interactions between polymers and inorganic nanoparticles are illustrated in Figure 5a. Fillers interact with anions as cross‐linked centers for polymer segments;^[^ ^17^ ^]^ therefore, interactions between anions and Li‐ions are reduced, which facilitates the Li‐salt dissociation and restricts the anion mobility.^[^ ^92^ ^]^

**Figure 6:**
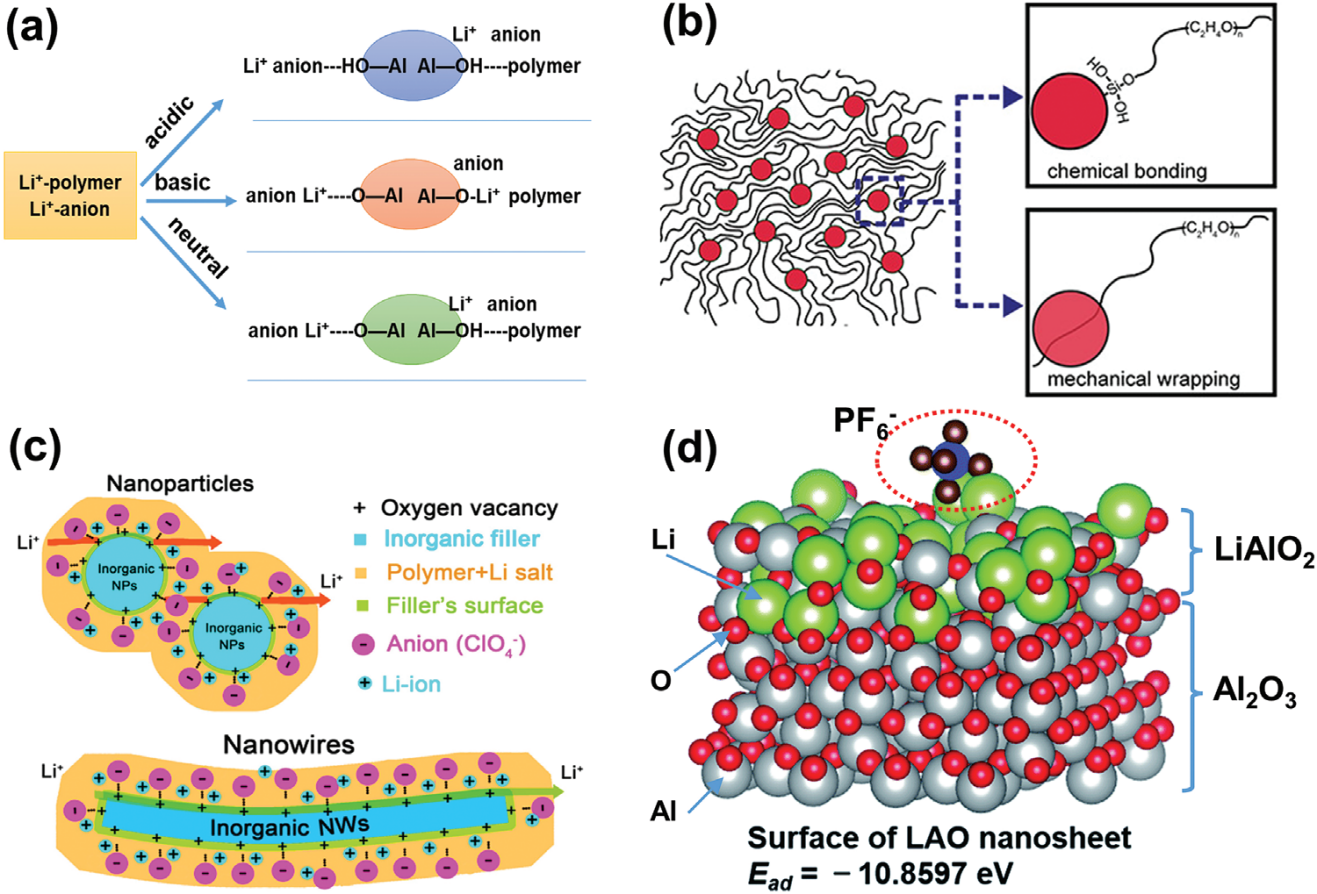
a) Pictorial model of surface interactions between three forms of dispersed fillers. Reproduced with permission.^[^ ^87^ ^]^ Copyright 2003, IOP Publishing. b) Schematic representation of Lewis acid–base interactions between polymer and SiO_2_ nanoparticles. Reproduced with permission.^[^ ^88^ ^]^ Copyright 2016, American Chemical Society. c) Schematic illustration of the Li‐ion transport in composite polymer electrolyte owing to the Lewis acid–base effect. Reproduced with permission.^[^ ^89^ ^]^ Copyright 2016, American Chemical Society. d) LiAlO_2_‐coated Al_2_O_3_ particles strengthening Lewis acid–base interactions. Reproduced with permission.^[^ ^90^ ^]^ Copyright 2021, Royal Society of Chemistry.

The Lewis acid–base effect is dependent on properties of added fillers, as explained in **Figure** **6**a. Three types of Al_2_O_3_ fillers, that is, acidic, basic, and neutral, played different roles in polymers. When acidic nanostructured‐Al_2_O_3_ was added, the polarizability of H^+^ ions in the acidic groups was stronger than that of the Li^+^ ions toward the polymer, and the affinity of anions toward the surface acidic groups of Al_2_O_3_ was higher than that of cations; both effects helped to separate Li^+^‐anion pairs.^[^ ^87^ ^]^ For Al_2_O_3_ with Lewis basic surface groups, interactions between the polar O atoms of Al_2_O_3_ and Li^+^ ions helped to dissociate both Li^+^‐anion pairs and polymer‐Li^+^ bonds, which resulted in higher concentration of free anions. Li^+^ ions interacted with polar O atoms via transient hydrogen bonding, and could migrate in the vicinity of the fillers.^[^ ^83^, ^87^ ^]^ On addition of neutral nanostructured‐Al_2_O_3_, both the aforementioned interactions occurred, but anions re‐associated with Li‐ions to form new ion pairs, which lowered the charge‐carrier concentration.^[^ ^4c^ ^]^ However, the Lewis acid–base model is largely explanatory and cannot provide a quantitative description of the conductivity change of composite polymer electrolytes with varying filler content due to missing geometric considerations.

Generally, materials with high dielectric constant or highly concentrated defects (e.g., oxygen vacancies or Li vacancies) demonstrate strong Lewis acid–base properties.^[^ ^93^ ^]^ Numerous publications report strategies to strengthen the Lewis acid–base interactions;^[^ ^94^ ^]^ some typical systems are given in **Table** **2**. Cui's group^[^ ^88^ ^]^ reported the fabrication of composite polymer electrolytes via in situ synthesis of monodispersed SiO_2_ nanospheres in PEO (Figure 6b). Strong PEO/SiO_2_ Lewis acid–base interactions enhanced the ionic conductivity. Cui et al.^[^ ^89^ ^]^ used Y_2_O_3_‐doped ZrO_2_ (YSZ) nanowires to generate high O‐vacancy concentration (Figure 6c). Positively charged O‐vacancies in YSZ were strong Lewis acid sites, which facilitated the LiClO_4_ dissociation and released free Li ions; consequently, the composite exhibited an ionic conductivity of 1.07 × 10^−5^ S cm^−1^ at 30 °C and a high Li^+^ transference number of 0.56, both of which were much higher than those of filler‐free polymer electrolytes. Some active fillers, for example, nano‐structured LLZO, showed stronger Lewis acid–base character because negatively charged Li^+^ vacancies created by intentional substitution acted as strong Lewis base centers.^[^ ^93^ ^]^ Nan et al.^[^ ^94a^ ^]^ triggered synergistic coupling between Li_6.75_La_3_Zr_1.75_Ta_0.25_O_12_ (LLZTO) and PVDF to strengthen acid–base interactions among PVDF, LiClO_4_, and LLZTO, which significantly improved the ionic conductivity. It should be noted that active fillers can induce extra and more complicated effects to influence the ionic conduction.

**Table 2:** Composite polymer electrolytes with different fillers

Filler	System	Conductivity [S cm^−1^]	Transference number	Ref.
Al_2_O_3_	PEO/LiClO_4_	1 × 10^−5^ @30 °C (Initial: 10^−6^)	0.31 (Initial: 0.16)	[95]
Gd_0.1_Ce_0.9_O_1.95_/La_0.8_Ga_0.8_Mg_0.2_O_2.55_ (full with oxygen vacancies)	PEO/LiTFSI	1.9 × 10^−4^ @30 °C (Initial: 10^−6^)	0.26 (Initial: 0.13)	[94b]
Nanosized Al_2_O_3_ with oxygen vacancies	PEO/LiTFSI	3.81 × 10**^−4^** @70 °C (Initial: 10^−5^)	0.27 (Initial: 0.27)	[96]
Monodispersed SiO_2_	PEO/LiClO_4_	4.4 × 10**^−^** ^5^ @30 °C (Initial: ≈3 × 10^−7^)	—	[88]
Palygorskite nanowires ((Mg,Al)_2_Si_4_O_10_(OH))	PVDF/LiClO_4_	1.2 × 10^−4^ @40 °C	0.54 (Initial: 0.54)	[75]
AlF_3_‐modified anodized aluminum oxide	PEO/LiTFSI	5.82 × 10^−4^ @30 °C (Initial: 3 × 10^−7^)	—	[16b]
Y_2_O_3_‐doped ZrO_2_ nanowires (Highly concentrated oxygen vacancies)	PAN/LiClO_4_	1.07 × 10^−5^ @30 °C (Initial: 2.1 × 10^−7^)	0.56 (Initial: 0.56)	[89]
Mg_2_B_2_O_5_ nanowires	PEO/LiTFSI	1.53 × 10^−4^ @40 °C (Initial: ≈1 × 10^−5^)	0.44 (Initial: 0.44)	[97]
Ta‐doped LLZO	PVDF/LiClO_4_	5 × 10^−4^ @25 °C (Initial: ≈7 × 10^−5^)	—	[94a]

Fillers modified with strong Lewis acid were also used in composite electrolytes to induce intense Lewis acid–base interactions at ceramic–polymer interfaces. For example, Guo and co‐workers^[^ ^90^ ^]^ introduced Al_2_O_3_ coated with strong Lewis acidic LiAlO_2_ into PVDF. Density functional theory (DFT) calculations in Figure 6d indicate significantly improved interactions between modified fillers and PF_6_^−^ anions from LiPF_6_ dissolved in PVDF; corresponding adsorption energy was calculated to be −10.86 eV. Consequently, the Li‐ion transference number was increased to 0.92. Similar effect was also reported by Cui et al.,^[^ ^16b^ ^]^ who used AlF_3_‐modified anodic aluminum oxide to strengthen the Lewis‐acid interaction.

#### 3.2.2 Space‐Charge Effect

**Figure 7:**
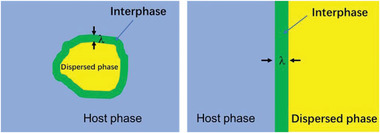
Space‐charge region in composite polymer electrolyte.

Recently, experimental results have shown that the ionic conductivity enhancement is closely related to space‐charge regions in polymer/active‐filler composites.^[^ ^86b^ ^]^ The introduction of secondary conducting‐phases breaks the thermodynamic equilibrium of defects, causing alterations in the conduction pathway. Discontinuity at the interfaces between the host and the dispersed phase causes deviations from the local electroneutrality and formation of a narrowly‐charged zone, which is normally labeled as “space‐charge region”, in which the concentrations of charge‐carrying defects (ionic and electronic defects) deviate from the bulk values, as illustrated in **Figure** **7**.

The full significance of the space‐charge region with respect to the ionic conductivity was first recognized by Liang,^[^ ^98^ ^]^ who conducted a systematic study of the electrical properties of the LiI/Al_2_O_3_ two‐phase system, and reported an anomalously high ionic conductivity, as compared with those of the two corresponding pure phases. As Al_2_O_3_ is electrically insulating, the conductivity enhancement was attributed to the space‐charge region at the LiI/Al_2_O_3_ interfaces. The space‐charge concept to explain the ionic conduction is strongly supported by two facts: i) The effects are boundary layer phenomena, while impurity effects, particularly the homogeneous doping, have been ruled out; ii) effective activation enthalpy for the enhanced conductivity is very similar to the enthalpy for the bulk ion migration in the cited examples. The space‐charge effect typically occurs at: i) ionic conductor/insulator interface (MX/A),^[^ ^99^ ^]^ ii) interface between two different ionic conductors (MX/MX’),^[^ ^100^ ^]^ and iii) grain boundaries in polycrystalline MX/MX.^[^ ^101^ ^]^ The space‐charge effect has been reviewed in several seminal works by Maier,^[^ ^85^ ^]^ who systematically studied the space‐charge effect in composite electrolytes, including metal cation conductors (e.g., Ag ions and Li ions).

The space‐charge effect has double influences on the ionic conduction. It provides a new kinetic pathway, and/or influences the ionic conductivity by affecting the point defect concentration in adjacent boundary zones. Motivated by the space‐charge effect, numerous studies have been conducted to optimize the ionic conductivity. Maier's group reported increase in the Ag‐ion conductivity by using mesoporous Al_2_O_3_.^[^ ^102^ ^]^ In accordance with the development of nano‐electronics, Maier proposed the concept of nano‐ionics.^[^ ^103^ ^]^

Guo et al.^[^ ^10^ ^]^ investigated the formation of the space‐charge region in the PEO/LLZO system. The driving force (free energy) causes migration of Li ions (${\mathrm{Li}}_{\mathrm{Li}}^{\times }$) from regular LLZO lattice sites to surface sites (*V*_s_) once LLZO nanoparticles are in contact with PEO (host phase), leading to aggregation of positively charged ions ($Li_{s}^{•}$) on the LLZO surfaces and negatively charged vacancies (*V*'_Li_) in the LLZO lattice. The defect reaction in the PEO/LLZO composite is:

(8) $${\mathrm{Li}}_{\mathrm{Li}}^{\times }+V_{s}⇌{\mathrm{Li}}_{s}^{•}+{V^{\prime }}_{\mathrm{Li}}$$

The system reaches a new equilibrium state after the migration process, creating a high Li‐ion concentration in the interfacial space‐charge region.

**Figure 8:**
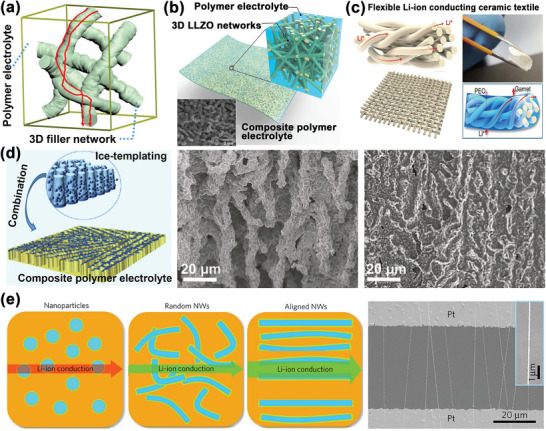
a) 3D LLZO skeleton providing continuous pathways for the Li‐ion conduction in composite polymer electrolyte. Reproduced with permission.^[^ ^78a^ ^]^ Copyright 2018, American Chemical Society. b) Schematic representation of 3D LLZO nanofiber network in composite polymer electrolyte. Reproduced with permission.^[^ ^105a^ ^]^ Copyright 2016, the National Academy of Sciences. c) Schematic representation of composite polymer electrolyte reinforced by solid‐garnet‐textile. Reproduced with permission.^[^ ^105b^ ^]^ Copyright 2018, Elsevier. d) Composite polymer electrolyte reinforced by vertically aligned LAGP‐ceramic. Reproduced with permission.^[^ ^15a^ ^]^ Copyright 2019, Elsevier. e) Composite polymer electrolyte reinforced by well‐aligned LLTO‐nanowires. Reproduced with permission.^[^ ^105c^ ^]^ Copyright 2017, Springer Nature.

The space‐charge effect is also used to strategically optimize the ionic conductivity. There are numerous publications on the space‐charge effect in composite polymer electrolytes.^[^ ^104^ ^]^ Fillers with various geometries, including 3D and well‐aligned fillers, were designed to improve the ionic conduction, as shown in **Figure** **8**.^[^ ^15^, ^78^, ^105^ ^]^ Guo's group^[^ ^75a^ ^]^ used 3D garnet frameworks as fillers (Figure 8a), Hu and co‐workers^[^ ^105a^ ^]^ synthesized different 3D filler‐frameworks, including 3D garnet nanofibers (Figure 8b) and 3D garnet textile (Figure 8c); the well‐sintered ion‐conductive networks not only conducted Li‐ions along the bulk phase but also provided additional expressways for the Li‐ion conduction in the more continuous space‐charge regions. In addition, well‐aligned fillers eliminate the low‐conducting crossing junctions and shorten the Li‐ion transport distances. Wang et al.^[^ ^15a^ ^]^ reported a composite polymer electrolyte reinforced by vertically‐aligned Li_1.5_Al_0.5_Ge_1.5_(PO_4_)_3_ (LAGP) ceramic fillers (Figure 8d); Cui et al.^[^ ^105c^ ^]^ synthesized well‐aligned LLTO‐nanowires for composite polymer electrolytes (Figure 8e). The aligned nanostructures were optimal for forming continuous space‐charge regions at the filler/polymer interfaces. Enhanced ionic conductivities derived from the space‐charge effect are summarized in **Table** **3**.

**Table 3:** Composite polymer electrolytes with active fillers

Filler	System	Conductivity [S cm^−1^]	Transference number	Ref.
Ta‐doped LLZO	PEO/LiTFSI	1.12 × 10^−5^	0.38	[75b]
3D LLZO	PEO/LiTFSI	1.2 × 10^−4^	0.33	[75a]
3D LLZO nanofibers	PEO/LiTFSI	1.12	0.52	[105a]
3D LLZO textile	PEO/LiTFSI	7 ×10^−5^	—	[105b]
3D LLZO nanofibers	PEO/LiTFSI	3.2 × 10^−4^	0.33	[104]
LLTO nanofibers	PAN/LiClO_4_	2.4 × 10^−4^	—	[76]
3D LLZO	PEO/LiTFSI	8.5 × 10^−5^	—	[15b]
3D LLTO	PEO/LiTFSI	8.8 × 10^−5^	—	[19]
Vertically‐aligned LAGP	PEO/LiTFSI	1.67 × 10^−4^	0.56	[15a]
Vertically‐aligned LATP	PEO/LiTFSI	5.2 × 10^−5^	—	[104]
Well‐aligned LLTO‐nanowires	PAN/LiClO_4_	6.05 × 10^−5^	0.42	[105c]

### 3.3 Modification of Local Structures in Polymer Hosts

Fillers may significantly change polymer structures due to the large structural mismatch and sudden change in chemical potential; therefore, structural modification or chemical reactions in polymer hosts and/or fillers may happen; such changes may have important influence on the ionic conductivity. In addition to the interfacial interactions, it is widely accepted that the addition of inorganic fillers can modify local structures of polymer chains by decreasing their crystallinity and glass‐transition temperature.^[^ ^84^ ^]^

In polymers, the ionic conduction predominantly occurs in amorphous regions through the local segmental motion. Therefore, low crystallinity enhances the ionic conduction. Thus, addition of fillers disrupts the polymer crystallinity to increase the amorphous character, facilitating the ionic mobility. In composite polymer electrolytes, dispersed secondary phase alters the phase stability of the polymer host as well as its microstructure, and increases the polymer‐segment hybridization; thus, decreasing and even eliminating the polymer crystallinity; therefore, significantly affecting the relaxation and conformation of the molecular chains. Scrosati et al.^[^ ^95^ ^]^ studied various PEO‐based composite polymer electrolytes with different inorganic nanofillers, reporting consistent decrease in *T*_g_ and corresponding decrease in the PEO crystallinity upon addition of inorganic fillers (*θ*‐Al_2_O_3_).

However, it remains unclear if it is cations (e.g., Li^+^) or anions (e.g., TFSI^−^) that move freely in the electrolyte once the crystallinity is decreased. Very often, the cation transference number is neglected. PEO is mostly used in solid polymer composite electrolyte systems; its ion transport mechanism has been known to involve cations (Li^+^) coordinating with oxygen atoms along the PEO polymer chains and diffusing via inter‐chain or intra‐chain hopping. On the basis of this understanding, the amorphous region created by fillers in the PEO electrolyte actually provides free space for anions (e.g., TFSI^−^) to move; therefore, the PEO‐based composite electrolyte normally shows a low cation transfer number;^[^ ^4b^ ^]^ the highest cation transference number of the highest ionically conductive composite (12.6 vol% LLZO in PEO) is only 0.42.^[^ ^86b^ ^]^

### 3.4 Ionic Conduction Pathways

Detailed ionic conduction pathways remain unclear. First, polymer chains are anchored onto surface sites of inorganic fillers through physical and/or chemical interactions, creating amorphous‐rich areas in polymers for rapid Li‐ion transport. Second, strong Lewis acid–base interactions and the space‐charge effect induced by inorganic fillers and ionic species in the polymer matrix increase the concentration of free Li‐ions, generating Li‐ion‐conductive substructures at the interfaces. Third, the Li‐ion conductive fillers act as the conduction pathways. Though detailed pathways are still under debate, various models and experiments have been developed, which successfully describe, even quantitatively in some cases, the ionic conduction in composite polymer electrolytes, as listed in **Table** **4**.

**Table 4:** Ionic conductivities and dominating conduction pathways in various composite polymer electrolytes

Composite	Filler content	Conductivity [S cm^−1^]	Dominating pathway	Ref.
PEO/LLZO	10 wt%	—	PEO matrix	[106]
PEO/LLZO	16 vol%	7.2 × 10^−5^	Interphase	[10]
PAN/LLZO	12.6 vol%	2.1 × 10^−4^	Interphase	[86b]
PEO/LLZO	50 wt% (20 vol%)	—	LLZO phase	[13]
PEO/LLZO	5 wt% 20 wt% 50 wt%	1 × 10^−5^ ≈1.4 × 10^−5^ 0.75 × 10^−5^	PEO matrix PEO matrix LLZO phase	[107]
PEO/LLZO	<10 wt% 10 wt% 50 wt% 80 wt%	≈4 × 10^−5^ ≈9 × 10^−5^ ≈3 × 10^−5^ ≈1 × 10^−5^	PEO matrix Interphase Interphase/LLZO phase LLZO phase	[108]
PEO/LLZO	0 wt% 20 wt% 50 wt% 80 wt%	≈3.5 × 10^−6^ ≈2 × 10^−4^ ≈4.5 × 10^−5^ ≈3.2 × 10^−4^	PEO matrix Interphase Interphase/LLZO phase LLZO phase	[109]
PAN/LGPS	70 wt%	2.2 × 10^−4^	Interphase	[110]
PAN/LLZO	5 wt%	1.31 × 10^−4^	Interphase	[93]
PEO/LLTO	15 wt%	2.4 × 10^−4^	Interphase	[79]
PEO/3D LLTO	≈45 wt%	8.8 × 10^−5^	Interphase	[111]
PEO/3D LLZO	≈40 wt%	1.2 × 10^−4^	Interphase/LLZO phase	[78a]

**Figure 9:**
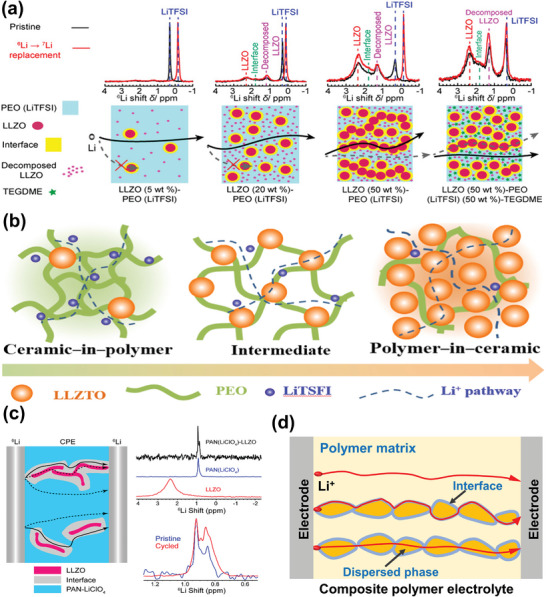
a) ssNMR spectra and schematic of Li‐ion pathways in PEO/LLZO (5 wt%), PEO/LLZO (20 wt%), PEO/LLZO (50 wt%), and TEGDME/PEO/LLZO (50 wt%) composite polymer electrolytes. Reproduced with permission.^[^ ^107^ ^]^ Copyright 2018, American Chemical Society. b) Illustration of Li‐ion conduction pathways in the PEO‐LLZTO composite, from “ceramic‐in‐polymer” to “polymer‐in‐ceramic”. Reproduced with permission.**^[^** ^108^ **^]^** Copyright 2018, Elsevier. c) ssNMR spectra and schematic of Li‐ion pathways in PAN/LLZO composites. Reproduced with permission.**^[^** ^93^ **^]^** Copyright 2018, American Chemical Society. d) Illustration of ion‐conducting pathways in composite polymer electrolyte.

In a very recent work on the Li‐ion transport mechanism in the LLZO/PEO composite by Zagórski et al.,^[^ ^106^ ^]^ the total Li‐ion conductivity was found to be governed by the polymer matrix rather than by the interface and the LLZO ceramics. In contrast, experimental and simulation results of Guo's group showed that the enhancement of the ionic conductivity was closely related to the space‐charge region at the two‐phase interface (Figure 5c).^[^ ^10^ ^]^ These results were also proven in another work by Guo and co‐workers;^[^ ^86b^ ^]^ they studied the size effect of the LLZTO filler, showing that the percolation threshold decreased when a filler with a smaller particle size was used. Hu and co‐workers^[^ ^107^ ^]^ employed solid‐state NMR to investigate the ion transport pathway in LLZO‐PEO composite electrolytes, and found that the ion transport gradually transformed from PEO to interfaces, and further, to loosely connected LLZO particles (**Figure** **9**a). Nan et al.^[^ ^108^ ^]^ synthesized composite electrolytes from “ceramic‐in‐polymer” (CIP) to “polymer‐in‐ceramic” (PIC) with different garnet‐particle contents. CIP electrolytes exhibited higher ionic conductivities than PIC electrolytes, while the latter had smaller activation energy than the former. These differences were mainly caused by different Li‐ion transport pathways, as illustrated in Figure 9b. In cases of low LLZO content, the conduction was mainly PEO mediated, while the LLZO/PEO interfaces and the LLZO body influenced the ion transport only beyond a threshold value of the LLZO content.

The characteristics of fillers and polymers also affect the ionic conduction in composite polymer electrolytes. Hu et al.^[^ ^93^ ^]^ prepared a PAN/LLZO composite and probed the ionic conduction pathways using ssNMR, discovering that Li‐ions preferred to travel through the LLZO/PAN interphases (Figure 9c), which was different from their previous work.^[^ ^13^ ^]^ They attributed the different results to the using of PAN as host; PAN required little LLZO to modify the interfacial conduction and improve the ionic conductivity. Hu et al.^[^ ^110^ ^]^ further studied the ionic conduction in LGPS/PEO composites via ssNMR, proving that the ionic conduction was mainly through the LAGP/PEO interface. They attributed this difference to the filler characteristics; rigid fillers could not closely integrate with the polymer matrix to form highly conductive interfaces. In contrast, soft fillers with large specific surface easily maximized the ionic conduction at interfaces, leading to improved ionic conductivity.

It is believed that ionic conduction pathways are related to the size, morphologies, and content of fillers. Differences between experiments and simulations or/and different experiments may be ascribed to the characteristics of fillers and polymers. Recent studies indicate multiple channels for the ionic conduction in composite polymer electrolytes. Many polymer–inorganic composites may have multiple conductive components, that is, the polymer matrix, the inorganic fillers, and the highly conductive interphases. Therefore, several migration processes are involved, including the ion transport within host materials, along dispersed phase–host interfaces and across dispersed phases,^[^ ^112^ ^]^ as illustrated in Figure 9d.

## 4 Quasi‐Solid/Gel Polymers

### 4.1 Overview

Quasi‐solid/gel polymers are a close variation of polymers, in which small‐molecule solvents function as plasticizers or the main dissolution agents for salts, showing a high ionic conductivity of >10^−3^ S cm^−1^. Compared with liquid electrolytes, quasi‐solid/gel polymers exhibit excellent mechanical properties (good strength, flexibility, etc.) and enhanced safety; compared with solid polymer electrolytes, quasi‐solid/gel polymers possess the merits of high ionic conductivity and superior electrode/electrolyte interfacial properties of liquid electrolytes. In general, quasi‐solid/gel polymer electrolytes are composed of polymer matrices, liquid solvents as plasticizers, Li salts, and additives such as inorganic fillers. PEO, PAN, PVDF, poly(vinylidene fluoride‐co‐hexafluoropropylene) (PVDF‐HFP), and PMMA have been commonly employed as polymer matrices for quasi‐solid/gel electrolytes. Plasticizers preferentially include carbonates (propylene carbonate, ethylene carbonate, dimethyl carbonate, and diethyl carbonate), ethers (tetraethylene glycol dimethyl ether, 1,2‐dioxolane and dimethoxymethane [DME]), succinonitrile, amides (*N*,*N*‐dimethylformamide [DMF]), sulfones (dimethyl sulfoxide [DMSO]), and ionic liquids,^[^ ^113^ ^]^ of which, polymers gelled with ionic liquids are also called as ionogel electrolytes.^[^ ^114^ ^]^

The small‐molecule solvents in the quasi‐solid/gel polymers influence the ionic conductivity in two important ways: First, they decrease the crystallinity and improve the polymer chain mobility; and second, they create liquid phases in the polymer. As noted previously, both liquid and polymeric ionic conduction mechanisms coexist in quasi‐solid/gel polymers. It is difficult to differentiate the contributions of the gelling liquid and the polymer because swollen polymer chains can carry substantial portions of ions, meaning that the nature of the solvent–polymer interactions and the morphology of the resulting gel are highly influential.

### 4.2 Ionic Conduction Occurs Through the Liquid Phase

**Figure 10:**
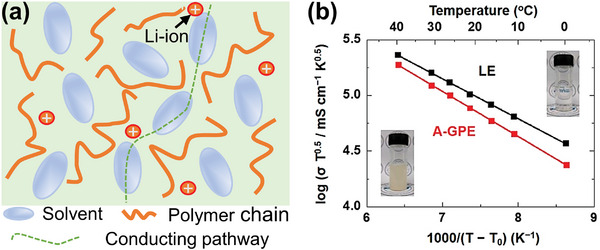
a) Schematic representation of typical quasi‐solid polymer and b) conductivities of liquid electrolyte and quasi‐solid electrolyte. Reproduced with permission.^[^ ^115^ ^]^ Copyright 2019, American Chemical Society.

It is widely accepted that the Li migration occurs primarily through the liquid phase as the Li‐ion transport in the solid phase is kinetically sluggish, while polymer chains serve as the inert mechanical framework (**Figure** **10**a). Kim et al.^[^ ^115^ ^]^ reported synthesis of acrylate‐based quasi‐solid polymers. The apparent activation energy for the ionic conduction (*E*_a_) in the quasi‐solid polymer, deduced from the slope of the VTF plots (Figure 10b), is quite similar to that of a liquid electrolyte, indicating no significant change in the ionic conduction mode. In addition, Chen and co‐workers^[^ ^116^ ^]^ suggested that the solvation structure and coordination number of Li^+^ are similar in the liquid electrolyte and the quasi‐solid electrolyte according to the Raman and NMR results. Their results demonstrate that the solvent molecules in the quasi‐solid electrolyte are free molecules and work for dissolving Li salts, just like in the liquid electrolyte, which forms a local high concentration of Li^+^ to ensure rapid ionic transport. Therefore, the liquid phase, rather than the polymer matrix, dominates the ionic conduction.

**Figure 11:**
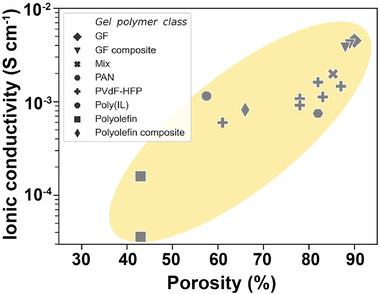
Ionic conductivity of several classes of gel‐polymer electrolytes versus porosity. A correlation between ionic conductivity and porosity is evident across classes of gel polymer electrolyte. Glass fiber (GF) composites are GF separators coated or impregnated with ionically conductive polymers. Reproduced with permission.^[^ ^47^ ^]^ Copyright 2021, Elsevier.

Given that liquids are broadly more conductive than polymers, pores among polymer chains influence the ionic conduction immensely; therefore, increasing the polymer porosity might be a straightforward way to improve the ionic conductivity. To test this assumption, the ionic conductivity is plotted against porosity for several materials (**Figure** **11**);^[^ ^47^ ^]^ a clear positive correlation is visible. As the relative contributions of the ionic conduction by the polymer phase and the liquid phase vary from one quasi‐solid electrolyte to another, the trend is not fully uniform. In addition, Guo's group^[^ ^117^ ^]^ reported a cross‐linked quasi‐solid polymer electrolyte with a high ionic conductivity of 4.3 mS cm^−1^; owing to the unique cross‐linked network, space between polymer chains induced high solvent uptake, thereby enabling the high ionic conductivity.

### 4.3 Ionic Conduction Via Polymer Chains and Immediately‐Surrounding Solvent

In at least some quasi‐solid/gel electrolytes; however, liquid‐filled pores are not major contributors to the bulk ionic conductivity; instead, the ionic conductivity is dominated by the conduction via polymer chains and the immediately‐surrounding solvent, which is different from the previous conduction mode (i.e., liquid phase dominating the ionic conduction). The change in the conducting model can be ascribed to the unique solvation structure of Li^+^ in quasi‐solid/gel polymer electrolytes (which is different from that of any known liquid): i) All the solvent molecules appear in the form of [solvent‐Li^+^] complexes; ii) polymer strongly interacts with the [solvent‐Li^+^] complexes, rather than with naked Li^+^ ions or solvents.

**Figure 12:**
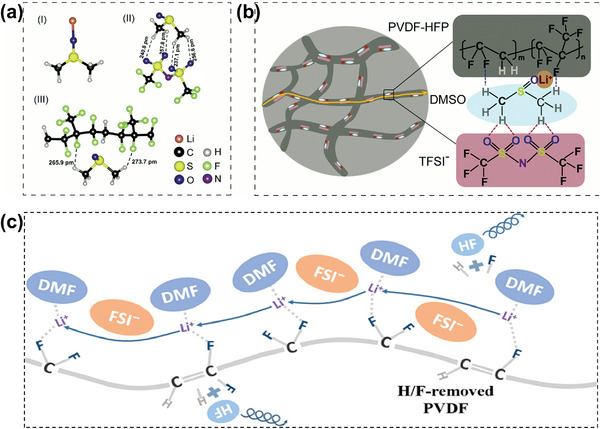
a) Optimized geometric configurations of the interaction systems of Li^+^/DMSO and b) Li^+^ transport model in the PVDF‐HFP/DMSO/LiTFSI‐based localized high‐concentration quasi‐solid polymer electrolyte. Reproduced with permission.^[^ ^118^ ^]^ Copyright 2022, Royal Society of Chemistry. c) Schematic illustration for the interactions and the lithium‐ transport in the PVDF/DMF/LiFSI quasi‐solid polymer electrolyte. Reproduced with permission.^[^ ^119^ ^]^ Copyright 2022, Elsevier.

Li's group^[^ ^118^ ^]^ reported a general localized high‐concentration strategy with the decoupling of the ion pairing and the ionic conduction (in a PVDF‐HFP/DMSO/LiTFSI based quasi‐solid polymer electrolyte). They suggested that PVDF‐HFP had minimal interaction with TFSI^−^, while the DMSO‐Li^+^ complex had stronger interaction with PVDF‐HFP (**Figure** **12**a). Therefore, the quasi‐solid polymer electrolyte was composed of particles with connection structures, in which DMSO acted as a bridge to rivet PVDF‐HFP and TFSI^−^. TFSI^−^ anions were anchored by this regulated structure, which inhibited the migration of TFSI^−^ and improved the Li^+^ transference number of the quasi‐solid polymer electrolyte. Multiple functional groups in the DMSO solvents (i.e., S=O of DMSO) act as Li^+^ binding sites to realize rapid Li^+^ transport in the particles of the electrolyte (Figure 12b). Therefore, polymer chains and the immediately‐surrounding solvent are responsible for the decoupling of ion pairing and ion conduction. A similar work of Wang's group^[^ ^119^ ^]^ confirmed that the polymer chain, the solvent, and the [solvent‐Li^+^] complex are closely intervolved in the ionic conduction of quasi‐solid polymer electrolytes, and their interactions make the polymer as the charge carrier host and the charge transport host, rather than a simple framework for liquid electrolytes. Figure 12c schematically shows the Li‐ion conduction mechanism in this quasi‐solid polymer electrolyte.

## 5 Conclusions and Perspective

Polymer‐based solid electrolytes, considered to be the most promising electrolytes for solid‐state batteries, have been extensively investigated. These materials will replace liquid electrolytes to minimize potential safety hazards and provide high‐energy‐density alternatives to current Li‐ion batteries. In this review, the ionic conduction mechanisms in polymer‐based electrolytes, including solvent‐free polymer electrolytes, composite polymer electrolytes, and quasi‐solid/gel polymer electrolytes are summarized and evaluated; relevant conduction models are discussed to gain insight into the ionic conduction. Nevertheless, numerous questions remain unanswered, and many issues remain unresolved; the scope for future research is immense.

Some critical topics envisioned for future research are:

(1) To increase the amount of the amorphous phase and to decrease the glass‐transition temperature *T*_g_ are effective to improve the ionic conductivity of polymer‐based electrolytes. However, low *T*_g_ often accompanies loss of mechanical properties in polymers. It is necessary to develop a novel polymer with enhanced ionic conductivity without compromising on the mechanical strength. Polymer molecular design (co‐polymerizing, cross‐linking, and grafting) could enable electrolytes with multiple functionalities; therefore, enhancing the ionic conductivity without sacrificing mechanical properties.

(2) Intermolecular chemistry of polymers requires further investigations. Polymer–electrolyte interactions (hydrogen‐bond, Lewis acid–base) promote the ion‐pair dissociation and the anion immobilization; thus, increasing the concentration of free Li ions and confining the anion motion. Interactions between polymers and other compounds should be investigated to increase the Li‐ion transference number as well.

(3) Interfacial interactions (Lewis acid–base effect and space‐charge effect) between fillers and host materials in composite polymer electrolytes are vital for achieving high ionic conductivities. More research works are required on underlying mechanisms of polymer/ceramic interfacial interactions and evolution. High‐volume manufacturing of composite polymer electrolytes with specific filler structures also requires investigation.

(4) Atomic‐level mechanism for the ionic conductivity enhancement needs to be determined. Numerous publications elucidate the ion diffusion in polymers using novel technologies (e.g., ssNMR). However, detailed knowledge of complex polymer systems, including interfacial diffusion in composite polymer electrolytes, is still lacking. Thus, it is necessary to develop dedicated experiments to investigate conductive behaviors. Advanced theoretical calculations and physical models have accelerated the electrolyte discovery and improved the fundamental understanding of the ionic conduction. Models with sufficient predictive power need to be developed to quantitatively evaluate the ionic conduction, including transport kinetics and pathways.

With the development of novel theoretical tools and advanced characterization techniques, it is reasonable to expect important breakthroughs in the understanding of the ionic conduction mechanisms in polymer‐based solid electrolytes. Studies on the ionic conduction could offer guidance for optimizing the ionic conductivity and promote fine‐tuning polymer‐based electrolytes with specific properties.

## Conflict of Interest

The authors declare no conflict of interest.
